# Comprehensive surveillance of MicroRNA to discriminate between muscle-invasive and non-muscle-invasive urothelial carcinoma based on noninvasive urinary small RNA sequencing in Taiwanese patients

**DOI:** 10.1186/s12885-025-15284-5

**Published:** 2025-11-22

**Authors:** I-Ning Yang, Chun-An Liang, Chia-Chun Wu, Qian-Sheng Xu, Shang-Wen Lin, Ming-Cheng Wang, Ming-Jenn Chen, Yuan-Shuo Hsueh, Hui Hua Chang

**Affiliations:** 1 Institute of Clinical Pharmacy and Pharmaceutical Sciences, College of Medicine, National Cheng Kung University, Tainan, Taiwan; 2 Division of Nephrology, Department of Internal Medicine, Chi Mei Medical Center, Tainan, Taiwan; 3 Department of Pharmaceutical Science and Technology, Chung Hwa University of Medical Technology, Tainan, Taiwan; 4 Department of Pharmacy, Kaohsiung Veterans General Hospital, Kaohsiung, Taiwan; 5 School of Pharmacy, College of Pharmacy, Kaohsiung Medical University, Kaohsiung, Taiwan; 6 Division of Nephrology, Department of Internal Medicine, National Cheng Kung University Hospital, College of Medicine, National Cheng Kung University, Tainan, Taiwan; 7 Department of Surgery, Chi Mei Medical Center, Tainan, Taiwan; 8 School of Post Baccalaureate Medicine, College of Medicine, Kaohsiung Medical University, Kaohsiung, Taiwan; 9 Center for Cancer Research, Kaohsiung Medical University, Kaohsiung, Taiwan; 10 Department of Medical Research, Kaohsiung Medical University Hospital, Kaohsiung, Taiwan; 11 School of Pharmacy, College of Medicine, National Cheng Kung University, Tainan, Taiwan; 12 Department of Pharmacy, College of Medicine, National Cheng Kung University Hospital, National Cheng Kung University, Tainan, Taiwan; 13 Department of Pharmacy, National Cheng Kung University Hospital, Dou- Liou Branch, Douliu, Yunlin, Taiwan

**Keywords:** MicroRNA, Urothelial cancer, Muscle-invasive urothelial carcinoma, Early detection

## Abstract

**Background:**

Muscle-invasive urothelial carcinoma (MIUC) poses higher risks of recurrence and metastasis compared with non-muscle-invasive urothelial carcinoma (NMIUC). However, difficulties in early detection and the invasive nature of current diagnostic procedures. Using next-generation sequencing (NGS), we aimed to profile comprehensive surveillance of urinary microRNAs (miRNAs) as a promising non-invasive tool for assessing urothelial carcinoma (UC) and identify differentially expressed miRNAs (DEmiRNAs) in patients with MIUC or NMIUC, providing insights into UC progression and molecular mechanisms in Taiwanese patients. We analyzed the clinical differences between patients with MIUC or NMIUC in treatment-naïve patients.

**Methods:**

Urinary miRNAs were profiled using NGS. DEmiRNAs were identified with DESeq2, and target prediction was performed using TarBase-v9.0 and DIANA-microT. Enrichment analysis was conducted with the miEAA tool to link DEmiRNAs to biological pathways and processes, focusing on the KEGG pathways and Gene Ontology (GO) annotations.

**Results:**

Among the 52 participants, significantly more patients with MIUC used herbal medicines compared with the NMIUC group (*p* = 0.027). A total of 1,967 DEmiRNAs were detected comprehensively, with 691 upregulated and 232 downregulated in MIUC versus NMIUC. Notably, hsa-miR-3168 exhibited the highest upregulation (10.2-fold), while hsa-miR-511-3p was the most downregulated (9.32-fold). KEGG analysis revealed that upregulated DEmiRNAs were most enriched in amino acid biosynthesis pathways, while downregulated DEmiRNAs were associated with the Hedgehog signaling pathway.

**Conclusions:**

This study highlights urinary DEmiRNAs detected via NGS as a potential non-invasive diagnostic tool. Pathway analyses of DEmiRNAs provided valuable insights into UC progression and offers opportunities for future therapeutic development.

**Supplementary Information:**

The online version contains supplementary material available at 10.1186/s12885-025-15284-5.

## Introduction

Urothelial carcinoma (UC) is a cancer that begins in the urothelial cells which line the lower (bladder and urethra) or upper urinary tract (pyelocaliceal cavities and ureter). UC is the predominant histologic type in developed countries, where it accounts for approximately 90% of bladder cancers [1]. Bladder cancer is the ninth most common type of cancer worldwide, with increasing rates of incidence and mortality [2]. Globally, more than 610,000 people were diagnosed with bladder cancer in 2022, and over 220,000 people died from the disease [2]. The UC progression is typically categorized by its invasion depth as muscle invasive (stage 2–4) or non-muscle invasive (stage 0–1). Approximately 70% of new cases of UC are classified as non-muscle invasive urothelial cancer (NMIUC) and 30% as muscle-invasive urothelial cancer (MIUC) or metastatic cancer [3]. MIUC has a higher risk of recurrence and metastasis compared with NMIUC, making early detection and treatment critical [4]. Known risk factors that precipitate UC include: smoking; *Schistosoma* infection; exposure to chemical elements, including arsenic and heavy metals [5]; and medications, such as cyclophosphamide and aristolochic acid [6].

In Taiwan, the annual incidence of UC is two- to three-fold greater than that reported globally, accompanied by an unusually high prevalence of UC in women [7–9]. The high incidence of UC in Taiwan is believed to originate from the abuse of aristolochic acid and arsenic contamination. Aristolochic acid is one of the main factors contributing to UC in China and Taiwan and has been banned in Taiwan since 2003 [10]. More than 90% of patients with UC in Taiwan exhibit aristolochic acid-associated somatic mutations, including aristolactam-DNA adducts, and more than 30% of them exhibit A: T-to-T: A transversion in the *TP53* gene [8]. Arsenic intoxication can lead to black foot disease and malignancies, including UC. A retrospective study from 1993 to 2006 revealed that patients with UC in arsenic-polluted areas located on the southwestern coast of Taiwan, including Chiayi (Budai and Yijhu) and Tainan (Beimen and Syuejia), with arsenic levels of approximately 350–1100 ng/ml in well water presented with a higher grade of UC and a lower cancer-specific survival rate than those in areas with drinking water with less or no arsenic [11]. Therefore, developing a diagnostic method for early detection and efficient follow-up of UC may be cost-effective.

Cystoscopy is the standard examination for the diagnosis and follow-up of UC [12]. However, cystoscopic examination may impact patients’ quality of life and is cost-intensive, invasive, and operator-dependent [13, 14]. Liquid biopsy is a widely investigated strategy for cancer diagnosis other than tissue biopsy because of its noninvasiveness and convenience in obtaining biological samples, enabling potentially early diagnosis and follow-up [15, 16]. Several biomolecules, including circulating cells, DNAs, proteins, and noncoding RNAs, have been proposed as targets for liquid biopsies [16]. Among the noncoding RNAs investigated, microRNAs (miRNAs) account for the smallest population and are the most well-studied biological sources because of their high abundance and stability in biological fluids as well as their tissue specificity, which endows them with the ability to diagnose cancers with unidentified tissue sources [17, 18].

miRNAs are short single-stranded non-coding RNAs, which often consist of 19–25 nucleotides, that are involved in regulating the expression of several genes [19]. miRNAs have been widely investigated as a target for both cancer diagnosis and treatment. Several studies have indicated the unique expression of miRNAs associated with UC development [20]. Epithelial-mesenchymal transitions (EMTs)-related miRNAs have a vital role in tumorigenesis, invasion, and metastasis such as miR-141b, miR-200 s, or miR-20 [21]. Moreover, miR-214 and miR-222 were found to be down-regulated in invasive bladder samples [22]. Thus, analysis of the miRNA profile may provide insights into the molecular mechanisms involved in UC progression. Several studies have utilized miRNA microarray methods to detect miRNAs in urine [21, 22]. However, the low miRNA concentration in urine increases the difficulty of detection, and microarray methods limit the types of miRNAs that can be detected [23]. Furthermore, there are a lack of clues provided by comprehensive surveillance at the transcriptome level, and the current data for some miRNAs are controversial and inconsistent among independent studies [21–23].

Therefore, in this study, we enrolled patients with UC, profiled their urinary miRNA expression comprehensively by next-generation sequencing (NGS), and analyzed their putative targets. We aimed to distinguish key clinical factors and differentially expressed miRNAs (DEmiRNAs) between patients with MIUC and NMIUC. Furthermore, we aimed to advance the understanding of the molecular mechanisms underlying UC progression through pathway analysis of DEmiRNAs.

## Materials and methods

### Participant recruitment and sample collection

This study was conducted at Chi Mei Medical Center in southern Taiwan. The Institutional Review Board of Chi Mei Medical Center approved the study protocol (approval number: 10612-015). All participants provided written informed consent prior to enrollment in accordance with the Helsinki declaration. We included treatment-naive adult (≥ 20 years) patients who were first diagnosed with UC through cellular or other pathological examinations between February 13, 2018 and July 13, 2023. We excluded individuals without urine samples due to end-stage of renal disease. The urine samples were obtained prior to radical cystectomy. We also collected the baseline demographic characteristics, medical and social history, presence of comorbidities, clinicopathologic characteristics, and laboratory data of all participants.

Urine samples were collected from each participant and stored at 4 °C until processing. The samples were first centrifuged at 2,000 g for 15 min. Then, the resulting supernatants were transferred to new tubes and centrifuged again at 12,000 g for 10 min at 4 °C. The final aliquots were stored at − 80 °C until use. The miRNeasy Mini Kit (Qiagen) was used to extract RNA from these specimens, following the manufacturer’s instruction. The RNA was eluted with 14 µL RNase-free water and a Qubit^®^ Fluorometer with a Qubit^®^ microRNA Assay Kit were used to quantify all samples. Fragment analysis was conducted using the Fragment Analyzer 5200 in conjunction with the Agilent DNF-471 RNA Kit (15 nt).

### RNA library preparation and sequencing

The 5 uL total RNA which was extracted from the 2 mL urine samples was used as input material for the small RNA sample preparations. Sequencing libraries were generated using QIAseq^®^ miRNA Library Kit (QIAGEN, Germany), following the manufacturer’s recommendations. Briefly, 3’ and 5’ adaptors were directly and specifically ligated to the 3’ and 5’ end of small RNA, respectively. Then first-strand cDNA was synthesized using QIAseq miRNA NGS RT Enzyme and RT primer. After polymerase chain reaction amplification, the library was size-selected with 170–200 bp by QIAseq beads. The quality and quantity of purified libraries were assessed on the Qsep400 system (Bioptic Inc., Taiwan) and Qubit 2.0 Fluorometer (Thermo Scientific, Waltham, MA, USA). The qualified libraries were then sequenced on illumina NovaSeq x plus with trimmed 75 bp single-end reads generated by Genomics, BioSci & Tech Co., New Taipei City, Taiwan.

### MiRNA sequencing analyses (pre‑processing of raw data and analysis)

The nf-core/rnaseq pipeline, one of the most utilized pipelines in the nf-core portfolio, is used for data pre-processing and quality control, and offers multiple alignment and quantification routes [24]. The RNA-seq pipeline of nf-core framework (v.3.3) was launched with Nextflow (v.21.04.0) to analyze RNA-seq data. Initial assessment of raw sequencing reads was performed using FastQC and miRTrace to evaluate data quality. The raw sequenced data was removed using TrimGalore! (v0.6.6), and mapped to reference genome, mature and hairpin miRNAs (miRBase v.22.1) with Bowtie (v1.3.0) to obtain proper miRNA reads. After alignment, SAMtools was utilized for additional processing of the aligned reads, generating basic statistics, and ensuring alignment accuracy. The counts of mature miRNA are analyzed using edgeR, with trimmed mean of M (TMM) values normalization applied to account for library size differences.

### Differential expression MiRNA analysis and MiRNA target prediction

To identify DEmiRNAs, all mature DEmiRNAs were identified using DESeq2 (v1.48.0) [25]. For group comparison, differential expression analysis filtered miRNAs with a q-value of less than 0.001 and a |log2 fold change| of more than 1 as significantly DEmiRNAs for subsequent analysis [26]. Log2-transformed fold change (log2FC) with positive value was taken as an up-regulated miRNA and the same with a negative value was taken as a down-regulated miRNA. TarBase-v9.0, high quality experimentally-supported miRNA targets on protein-coding region, and DIANA-microT 2023, utilizing information from Ensembl v102, miRBase 22.1, and MirGeneDB 2.1, were used for target prediction of DEmiRNAs [27, 28].

### MiRNA enrichment analysis

The DEmiRNAs between MIUC and NMIUC with a total count of five or more reads were uploaded and underwent miRNA enrichment analysis. The miRNA Enrichment Analysis and Annotation tool (miEAA) (https://ccb-compute2.cs.uni-saarland.de/mieaa2/) is an online tool used for miRNA enrichment analysis [29]. This tool is tailored for miRNA precursors and mature miRNAs in multiple species, such as humans, and connects miRNA expression patterns to biological functions and pathways. We performed the gene set enrichment analysis of DEmiRNAs from the miEAA database version 2.1, and selected “miRPathDB database” for identifying KEGG pathways (KEGG) and “Gene Ontology (GO) (miRWalk)” as the reference gene set for understanding biological processes [30].

### Statistical analysis

Categorical variables are presented as counts and percentages, while continuous variables are presented as means (standard deviations). To compare baseline patient characteristics between those with MIUC and NMIUC, the chi-squared or Fisher’s exact test were used for categorical variables and the one-way analysis of variance was used for continuous variables. Statistical analyses were conducted using Statistical Analysis Software version 9.4 for Windows (SAS Institute Inc., Cary, NC, USA). A *p*-value of less than 0.05 was considered statistically significant.

## Results

### Participant enrollment and characteristics

**Fig. 1:**
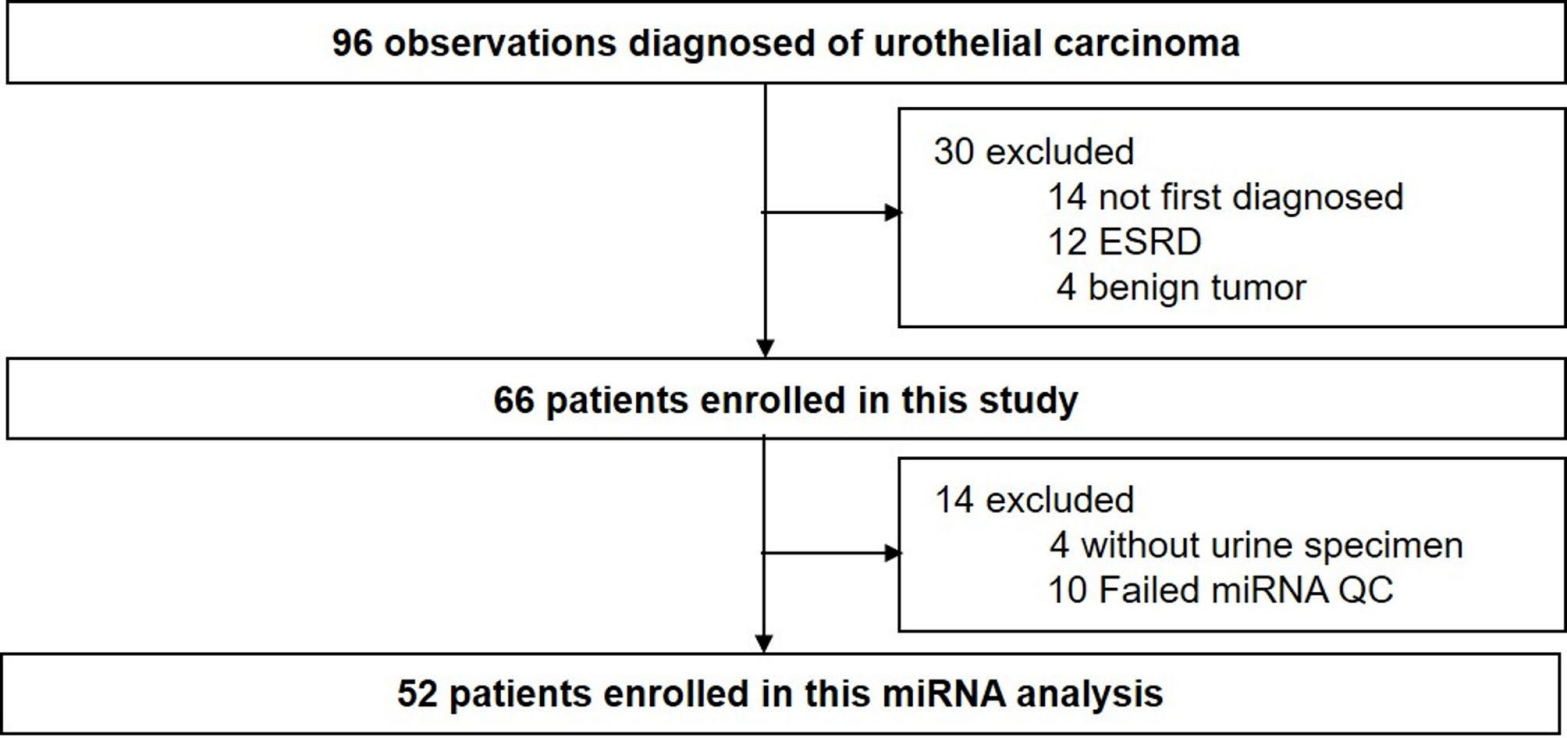
Participant enrollment. ESRD, end-stage renal disease; miRNA, microRNA

During the study period, 96 adult inpatients diagnosed with UC were screened for inclusion and 30 were excluded due to previously diagnosed UC (*N* = 14), end stage renal disease (*N* = 12), and benign tumor (*N* = 4). Among the 66 participants who met the inclusion criteria, miRNAs were extracted from 62 patient urine samples as four patients did not have urine specimens. Finally, 52 patients who passed our quality control filters for miRNA were enrolled for miRNA analysis (Fig. 1).

The mean participant age was 70 years and 34.6% were men. Furthermore, 53.8% of patients had a history of hypertension, 38.5% had diabetes mellitus, 11.5% had hyperlipidemia, 28.8% were current or former smokers, and 17.3% were alcoholic. Furthermore, 42.3% and 38.5% of the patients had a history of long-term non-steroidal anti-inflammatory drug and Chinese herbal medicine use, respectively. Table 1 shows the baseline characteristics, comorbidities, and medical history of patients with MIUC or NMIUC. Age, sex, most comorbidities, and social history were generally similar between the groups. Nearly half of the patients with MIUC had taken herbal medicine which was significantly higher than the number of patients in the NMIUC group (*p* = 0.027). The renal pelvis is the most common organ affected by UC, followed with the ureters and bladder. The mean number of organs affected by UC at diagnosis was 1.1 ± 0.4, without significant difference between those with MIUC or NMIUC. Most patients had chronic kidney disease stage 2 to 4 with slightly low albumin levels. Laboratory data were similar between the groups except for the hemoglobin level which was lower in the NMIUC group (*p* = 0.007).

**Table 1:** Participant characteristics

Variables	All (*n* = 52)	Muscle invasive UC (*n* = 37)	Non-muscle invasive UC (*n* = 15)	*p*-value
Male	18 (34.6)	13 (35.1)	5 (33.3)	1.000
Age (years)	70.0 ± 8.5	70.7 ± 8.3	68.3 ± 9.2	0.375
Height (cm)	157.3 ± 8.8	158.0 ± 7.9	155.5 ± 10.8	0.362
Weight (kg)	60.6 ± 12.1	60.6 ± 11.3	60.5 ± 14.3	0.968
Body mass index	24.4 ± 4.4	24.2 ± 4.0	24.8 ± 5.3	0.655

Comorbidity				
Hypertension	28 (53.8)	21 (56.8)	7 (46.7)	0.553
Diabetes mellitus	20 (38.5)	14 (37.8)	6 (40.0)	1.000
Hyperlipidemia	6 (11.5)	5 (13.5)	1 (6.7)	0.659
Smoker	15 (28.8)	12 (32.4)	3 (20.0)	0.506
Alcoholic	9 (17.3)	7 (18.9)	2 (13.3)	1.000
NSAID	22 (42.3)	15 (40.5)	7 (46.7)	0.762
Herbal medicine	20 (38.5)	18 (48.6)	2 (13.3)	0.027*

Cancer sites				
Ureter	20 (38.5)	12 (32.4)	8 (53.3)	0.213
Bladder	5 (9.6)	3 (8.1)	2 (13.3)	0.619
Renal pelvis	30 (57.7)	23 (62.2)	7 (46.7)	0.363
Number of organ involved	1.1 ± 0.4	1.0 ± 0.4	1.1 ± 0.4	0.409

Tumor pathology				
Size (cm)	4.4 ± 2.8	4.5 ± 2.9	4.1 ± 2.5	0.666
High grade malignancy	49 (96.1)	35 (94.6)	14 (100.0)	1.000
Lymphovascular invasion	17 (33.3)	15 (40.5)	2 (14.3)	0.102

Laboratory data				
eGFR (mL/min/1.73)	53.2 ± 22.3	51.7 ± 22.2	56.7 ± 23.2	0.474
Ca (mg/dL)	8.2 ± 1.0	8.2 ± 1.1	8.1 ± 0.8	0.779
P (mg/dL)	3.8 ± 1.0	3.9 ± 1.1	3.5 ± 0.9	0.180
Albumin (g/dL)	3.4 ± 0.5	3.4 ± 0.5	3.3 ± 0.5	0.359
Low-density lipoprotein (mg/dL)	97.5 ± 41.2	101.9 ± 42.9	86.7 ± 35.8	0.230
Total cholesterol (mg/dL)	160.4 ± 46.1	163.7 ± 49.2	152.1 ± 37.6	0.417
Triglyceride (mg/dL)	99.3 ± 43.0	100.6 ± 41.2	96.1 ± 48.5	0.738
HbA1c (%)	6.3 ± 0.9	6.3 ± 0.9	6.4 ± 1.0	0.586
CRP (mg/L)	32.0 ± 26.0	30.6 ± 26.5	35.9 ± 25.3	0.524
Hemoglobin (g/dL)	11.5 ± 1.6	11.8 ± 1.7	10.7 ± 1.0	0.007*

Data are presented as numbers (%) or means (standard deviations). Bold font indicates statistical significance

*Abbreviations:NSAID* Non-steroidal anti-inflammatory drugs, *UPCR* Urine protein and creatinine ratio,* eGFR* Estimated glomerular filtration rate, *HbA1c* Hemoglobin A1c, *CRP* C-reactive protein

^*^p value < 0.05

### Candidate MiRNAs and targeted genes

**Fig. 2:**
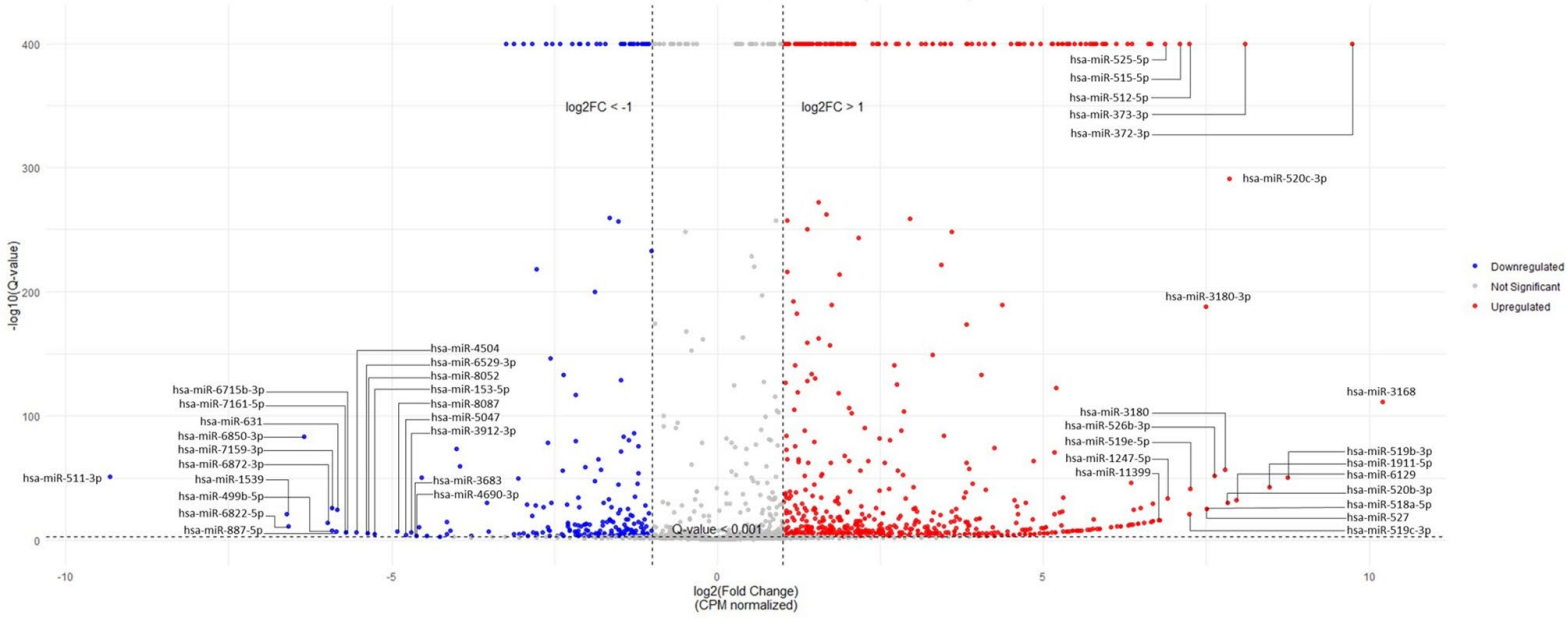
The differentially expressed miRNAs in patients with urothelial carcinoma. The top 20 significantly expressed upregulated and downregulated miRNAs in patients with MIUC compared with those with NMIUC are labeled in the figure. Significant differentially expressed miRNAs were defined as those with a q-value < 0.001 and a |log2 fold change| >1

A total of 1,967 miRNAs were detected in 52 urine samples, including 1,344 upregulated and 624 downregulated miRNAs. Compared with the urine samples from patients with NMIUC, the urine samples from patients with MIUC exhibited 691 upregulated and 232 downregulated DEmiRNAs (q-value < 0.001 and log 2 Fold - Change $\:\ge\:$ 1). The top 20 significantly expressed upregulated and downregulated miRNAs in patients with UC are listed in Fig. 2.

Then, we performed receiver operating characteristic (ROC) analyses to assess diagnostic accuracy in distinguishing MIUC from NMIUC. We first developed a logistic regression model using two significant clinical variables—herbal medicine use and hemoglobin level—which yielded an area under the curve (AUC) of 0.83 (Supplementary Table 1, Additional file 1; Supplementary Fig. 1, Additional file 2). Subsequently, we incorporated one significantly associated miRNA (hsa-miR-3180-3p, *p* = 0.025) into the model. The combined model achieved an improved AUC of 0.89 (Supplementary Fig. 1, Additional file 2), indicating that inclusion of miRNA expression enhanced the model’s discriminatory ability between MIUC and NMIUC. These findings support the potential of urinary miRNAs, particularly hsa-miR-3180-3p, as complementary biomarkers for non-invasive diagnosis.

Remarkably, hsa-miR-3168 was the most upregulated DEmiRNAs, with a 10.2-fold change compared with that of controls. The expression of other miRNAs (hsa-miR-372-3p, hsa-miR-519b-3p, hsa-miR-1911-5p, and hsa-miR-373-3p) was increased by more than eight‐fold compared with that of patients with NMIUC. Meanwhile, hsa-miR-511-3p was the most downregulated DEmiRNAs in patients with MIUC, with a −9.32‐fold change compared with those with NMIUC. Moreover, the expression of hsa-miR-1539, hsa-miR-6822-5p, and hsa-miR-6850-3p were -six‐fold lower in patients with MIUC than in those with NMIUC. Gene targets for the top 20 upregulated and downregulated DEmiRNAs were also determined through TarBase-v9.0 and DIANA-microT 2023 (Supplementary Tables 2–5, Additional file 1).

In TarBase V9.0, the top 20 upregulated DEmiRNAs, 2015 miRNA-gene interactions were reported. Among these, 13 strong miRNA-gene interactions (micro_tscore = 1) were identified, such as: hsa-miR-520c-3p with *TGFBR2*, hsa-miR-520b-3p with *MAP3K2*, *TGFBR2*, and *ZBTB20*. Similarly, among the top 20 downregulated miRNAs, 240 interactions with genes by high throughput direct experimental study have been reported. Among all the downregulated miRNA-gene interactions, hsa-miR-153-5p showed very strong interactions with the following four genes: *FAM171A1*, *N4BP2*, *STXBP5*, and *TCEAL7*, with a high predicted binding or regulatory confidence (micro_tscore = 1) [31, 32]. The interaction score of miRNAs and genes are presented in Supplementary Tables 4 and 5, Additional file 1.

### KEGG pathway enrichment analysis

**Fig. 3:**
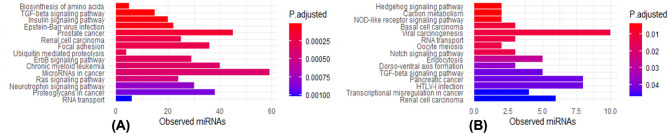
KEGG pathway analysis of differentially expressed miRNAs between the groups. The top 15 enriched pathways are presented (|log2 fold change| >1 and q-values < 0.001). **A** Numbers of upregulated functional differentially expressed miRNAs involved in each pathway; (**B**) Numbers of downregulated functional differentially expressed miRNAs involved in each pathway. Detailed enrichment statistics, including observed counts and adjusted p-values, are provided in Supplementary Data

The DEmiRNAs between patients with MIUC or NMIUC were evaluated using the miEAA tool to do the pathway enrichment analysis. In the KEGG pathway enrichment analysis, we found 77 pathways to be regulated by upregulated miRNAs and 16 to be regulated by downregulated miRNAs. The top three upregulated DEmiRNAs modulated the biosynthesis of amino acids, the TGF-beta signaling pathway, and the insulin signaling pathway (Fig. 3A). The top three significant pathways involved with the downregulated miRNAs included the Hedgehog (Hh) signaling pathway, carbon metabolism, and the NOD-like receptor signaling pathway (Fig. 3B**)**. Detailed enrichment statistics, including observed counts and adjusted p-values, are provided in Supplementary Tables 6 and 10, Additional file 1.

### GO analysis

**Fig. 4:**
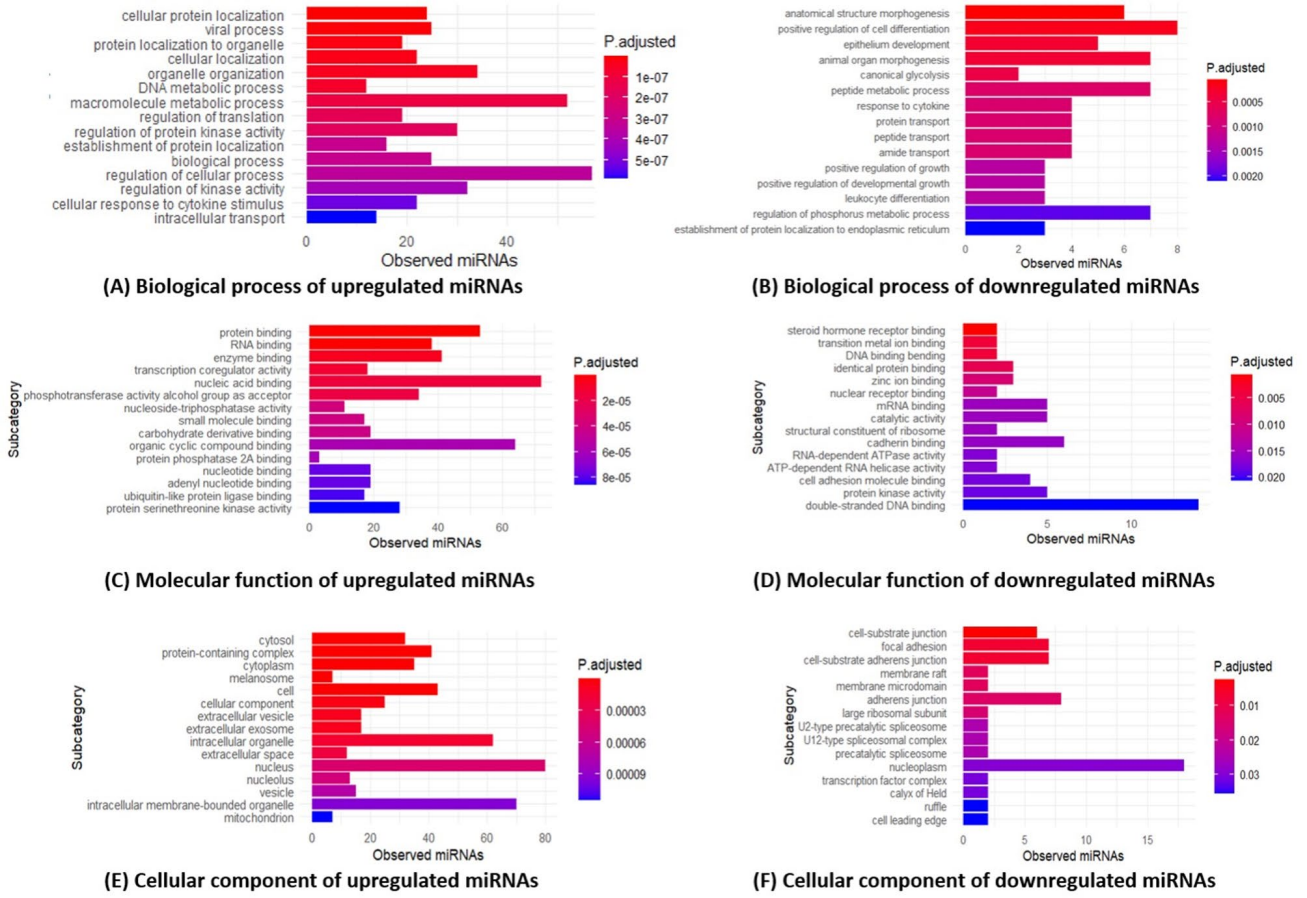
Gene ontology (GO) analysis of the biological process, molecular function, and cellular components, and annotations for the differential expression of miRNAs between the groups. The top 15 enriched pathways are presented (|log2 fold change| >1 and q-values < 0.001). Biological process (**A**), molecular function (**C**), and cellular component (**E**) enrichment analysis of the number of upregulated functional differentially expressed miRNAs. Biological process (**B**), molecular function (**D**), and cellular component (**F**) enrichment analysis of numbers of downregulated functional differentially expressed miRNAs. Detailed enrichment statistics, including observed counts and adjusted p-values, are provided in Supplementary Data

In our GO analysis, we found 1364 biological process to be modulated by upregulated DEmiRNAs and 216 by downregulated DEmiRNAs. The majority of upregulated DEmiRNAs were highly enriched in cellular protein localization, viral process, cellular localization, DNA metabolic process, organelle organization, and protein localization to organelles. In contrast, the top-ranked pathways for downregulated DEmiRNAs involved anatomical structure morphogenesis, positive regulation of cell differentiation, epithelium development, animal organ morphogenesis, and canonical glycolysis etc. (Fig. 4A and B).

Molecular function analysis provides deeper insights into the roles and locations of genes or gene products within a biological system, and focuses on the activities of gene products, such as protein binding, catalysis, or transport. We found molecular function to be associated with 147 upregulated and 33 downregulated DEmiRNAs. Protein, RNA, and enzyme binding were highly enriched molecular functions of upregulated DEmiRNAs. Downregulated DEmiRNAs were highly enriched in steroid hormone receptor binding, transition metal ion binding, and DNA binding bending (Fig. 4C and D).

The cellular component aspect of GO analysis describes the location within the cell or the part of the cell where a gene product is active or localized. Cellular component analysis revealed that upregulated DEmiRNAs were associated with 128 cellular components and highly enriched in the cytosol, protein-containing complex, and cytoplasm. In contrast, downregulated DEmiRNA were involved in 17 cellular components and highly enriched in cell-substrate junctions, focal adhesions, and cell-substrate adherens junctions (Fig. 4E and F). Detailed enrichment statistics, including observed counts and adjusted p-values, are provided in Supplementary Tables 7, 8, 9, 11, 12 and 13, Additional file 1.

## Discussion

This study profiles comprehensive surveillance of miRNAs and leverages target miRNAs as biomarkers to predict risk of progression to MIUC using noninvasive urine specimens as a direct and accessible sample source. The survival prognosis for patients with NMIUC is relatively favorable, with a 5-year overall survival of approximately 96% [31]. However, the 5-year survival rate for MIUC ranges between 40% and 70% depending on various factors such as stage, treatment options, and the patient’s overall health [33]. Therefore, the ability to predict progression risk to MIUC based on patient-specific characteristics and miRNA profile holds prognostic significance in clinical practice. Several clinical and pathophysiological factors are associated with poor prognosis such as multicentricity, largeness of the tumor, and tumor grade [34]. Likewise, in our study, patients with MIUC had a larger tumor size and a higher proportion of lymphovascular invasion compared with those with NMIUC. Furthermore, patients with MIUC had a higher proportion of herbal medicine use than those with NMIUC. Up to 39% of the Taiwanese population has been exposed to aristolochia-based herbal medicines over the past 30 years, which has contributed to a higher incidence of UC in Taiwan [35]. Hence, in 2003, Taiwan officially prohibited the import and sale of aristolochia-based herbal medicines.

In our dataset, the top 20 upregulated and downregulated urinary miRNAs at diagnosis differed from those identified in The Cancer Genome Atlas (TCGA) tumor tissue analyses. The Cancer Genome Atlas bladder urothelial carcinoma (TCGA-BLCA) dataset contains Illumina HiSeq–based miRNA profiles from 427 tumor tissues. In the TCGA-BLCA dataset (2 NMIUC and 425 MIUC cases), comparison between NMIUC and MIUC identified the top 20 most highly expressed miRNAs, including hsa-miR-490-3p, hsa-miR-1247-5p, and hsa-miR-204-5p (Supplementary Fig. 2, Additional file 2). The higher expression of hsa-miR-1247-5p in MIUC was consistent with our findings. However, most of the top 20 upregulated and downregulated miRNAs did not overlap with those showing the largest expression differences in the TCGA-BLCA dataset. This discrepancy may reflect differences in specimen type (noninvasive urine vs. resected tumor tissue), analytic focus (tumor invasiveness at presentation vs. overall survival/prognosis), population, or technical factors. Notably, our urine-based approach offers a clinically practical, low-risk method for early assessment of tumor aggressiveness, addressing a different clinical question from most TCGA-based reports.

In the KEGG analysis, a greater number of downregulated miRNAs in patients with MIUC were found to be involved in the Hh signaling pathway, leading to the enrichment of the Hh pathway. The Hh pathway plays an essential role in regulating cell growth, differentiation, and regenerate proliferation of epithelial stem cells in the bladder [32, 36]. The expression of the Sonic Hedgehog (Shh) protein, a member of the Hh family, is associated with lymph node invasion and metastasis in UC [37]. In their in-vitro and in-vivo experiments, Islam et al. demonstrated that the Shh protein induces EMT through activation of the TGF-β1 pathway. Shin et al. observed elevated levels of Shh and Gli1 mRNAs in response to bladder tissue injury, suggesting that Shh may promote the proliferative capacity of bladder stem cells, thereby contributing to cancer recurrence and resistance to therapy [36]. Furthermore, Chen et al. found that germ-line genetic variations in the Shh pathway predicted clinical outcomes in patients with NMIUC undergoing transurethral resection and BCG treatment [38]. The most significantly upregulated DEmiRNAs in patients with NMIUC compared with those with MIUC were those enriched in the biosynthesis of the amino acid pathway. Cancer cells frequently undergo alterations in amino acid metabolism to support the increased demands of rapid protein synthesis and cellular proliferation. Furthermore, metabolites derived from amino acids are integral to the regulation of epigenetic processes [39]. In UC, the upregulation of amino acid biosynthesis pathways may promote tumor growth and survival by ensuring a continuous supply of essential nutrients. Additionally, tumor cells, including those in UC, often shift towards aerobic glycolysis (the Warburg effect) as their primary energy source to sustain uncontrolled proliferation. Consequently, this elevated glycolytic activity is driven by the overexpression of genes involved in glycolysis, leading to the excessive production of metabolites such as pyruvate, alanine, and lactate [40].

In biological process analysis of GO, upregulated and downregulated DEmiRNAs are significantly enriched in the cellular protein localization and anatomical structure morphogenesis pathways, respectively. Cellular protein localization is crucial for maintaining proper cellular functions, including signaling, metabolism, and cell division. For example, mislocalization of tumor protein 53 (p53) in UC results in tumor progression and therapy resistance. Borowczak et al. found that high p53 expression, observed in metastatic tumors, resulted in shorter survival [41]. In UC, morphogenetic changes, particularly during EMT, can result in cancer cell migration, invasion, and metastasis. Loss or reduced expression of E-cadherin, maintaining cell-cell adhesion and epithelial integrity, is frequently observed in UC, leading to disrupted morphogenesis, EMT, and increased invasive potential of cancer cells [42].

In UC, protein binding enrichment and steroid hormone receptor binding pathways are crucial in regulating various molecular functions related to cancer progression, cell signaling, and tumor microenvironment interactions. The PI3K/AKT/mTOR pathway regulates key cellular functions and is dysregulated in UC. Targeting the PI3K/AKT/mTOR pathway offers a promising therapeutic strategy, especially in MIUC, with potential benefits when combined with chemotherapy or immunotherapy [43]. Several preclinical and clinical data also indicate that the steroid hormone receptor superfamily, including receptors for androgens, estrogen, glucocorticoids, progesterone, and vitamin D, play a critical role in the tumorigenesis of UC [44]. Inoue et al. proved that nuclear factor-κB facilitates urothelial tumorigenesis and cancer progression by interacting with androgen receptor signaling [45]. In the GO cellular component analysis, DEmiRNAs were enriched in the cytosol pathway and depleted in the cell-substrate junction pathway. As the cell-substrate junctions were depleted, particularly integrins, this resulted in disrupted cell adhesion and enhanced tumor invasiveness [46]. Integrins are key adhesion receptors that mediate interactions between the cell and extracellular matrix. In UC, alterations of integrin expression may result in enhanced adhesive behavior. Furthermore, integrins are involved in the development of metastasis and recurrence of UC [47].

Several limitations should be acknowledged in this study. First, we acknowledge the absence of an independent cohort due to limited sample availability. Second, we did not perform additional qPCR validation to confirm the reliability of sequencing results for selected DEmiRNAs. Third, experimental validation of the predicted miRNA–mRNA–protein interactions and associated pathway networks was not conducted. Fourth, DEmiRNA expression profiles were not analyzed in relation to patient outcome measures or potential confounding factors, such as herbal medicine use and prior exposure to aristolochic acid, which limits the evaluation of their prognostic significance. Fifth, advanced molecular approaches—including spatial transcriptomics, single-cell sequencing, and proteomics—were not employed, restricting insights into the cellular origins and downstream protein targets of the identified miRNAs. Addressing these methodological and analytical gaps in future studies will be essential to enhance the mechanistic understanding, translational potential, and clinical applicability of our findings.

## Conclusion

In conclusion, this study explored herbal medicine use in MIUC and NMIUC and identified a potential association with MIUC. Although prescriptions containing aristolochic acid have been banned in Taiwan since 2003, the long-term use reported by these patients raises the possibility of prior exposure. Moreover, we profiled comprehensive surveillance of miRNAs using NGS and identified DEmiRNAs in the urine samples of patients with NMIUC or MIUC, providing a potential non-invasive method for diagnosing MIUC. Additionally, our pathway analysis of DEmiRNAs may improve the current understanding of UC and open potential new avenues for anti-cancer therapy in further studies.

## Supplementary Information

Supplementary Material 1. Supplementary Figure 1. Receiver operating characteristic (ROC) curves for MIUC/NMIUC discrimination. The left panel shows the ROC curve of a logistic regression model including only clinical variables (herbal medicine use and hemoglobin level), with an area under the curve (AUC) of 0.83. The right panel shows the ROC curve of the model including both clinical variables and a significant urinary DEmiRNA (hsa-miR-3180.3p), achieving an improved AUC of 0.89. Supplementary Figure 2. Volcano plot of differentially expressed miRNAs between MIUC and NMIUC in the TCGA-BLAC dataset. Since the TCGA-BLAC dataset provides normalized miRNA expression values, the volcano plot shows an overall distribution shifted toward positive values. The left side represents miRNAs with relatively lower expression in MIUC, whereas the right side represents those with relatively higher expression in MIUC. The top 20 miRNAs with the largest expression differences compared to NMIUC, along with their Q-values, are shown.Abbreviations: MIUC, muscle-invasive urothelial carcinoma; NMIUC, non-muscle-invasive urothelial carcinoma; log₂FC, log₂(fold change); RPM, reads per million; Q-value, adjusted p-value.

Supplementary Material 2. Supplementary Table 1. Logistic regression analyses of DEmiRNAs for MIUC/NMIUC discrimination. Supplementary Table 2. List of top 20 significantly differentially expressed upregulated miRNAs and gene interaction in Tarbase. Supplementary Table 3. List of top 20 significantly differentially expressed downregulated miRNAs and gene interaction in Tarbase. Supplementary Table 4. List of top 20 significantly differentially expressed downregulated miRNAs and gene interaction in microT. Supplementary Table 5. List of top 20 significantly differentially expressed upregulated miRNAs and gene interaction in microT.

## Data Availability

The datasets generated and/or analyzed during the current study are available in the Zenodo repository, [https://zenodo.org/records/15228517](https://zenodo.org/records/15228517).

## Abbreviations

MIUC: Muscle-invasive urothelial carcinoma
NMIUC: Non-muscle-invasive urothelial carcinoma
NGS: Next-generation sequencing
miRNAs: MicroRNAs
UC: Urothelial carcinoma
DEmiRNAs: Differentially expressed mirnas
GO: Gene ontology
Shh: Sonic hedgehog
