# Supplementary material for: Comprehensive surveillance of MicroRNA to discriminate between muscle-invasive and non-muscle-invasive urothelial carcinoma based on noninvasive urinary small RNA sequencing in Taiwanese patients
Source: BMC Cancer. 2025 Nov 22;25:1900. doi: 10.1186/s12885-025-15284-5 (PMC12752029; doi:10.1186/s12885-025-15284-5)
Supplement: Supplementary file 1 — Supplementary Material 1. Supplementary Figure 1. Receiver operating characteristic (ROC) curves for MIUC/NMIUC discrimination. The left panel shows the ROC curve of a logistic regression model including only clinical variables (herbal medicine use and hemoglobin level), with an area under the curve (AUC) of 0.83. The right panel shows the ROC curve of the model including both clinical variables and a significant urinary DEmiRNA (hsa-miR-3180.3p), achieving an improved AUC of 0.89. Supplementary Figure 2. Volcano plot of differentially expressed miRNAs between MIUC and NMIUC in the TCGA-BLAC dataset. Since the TCGA-BLAC dataset provides normalized miRNA expression values, the volcano plot shows an overall distribution shifted toward positive values. The left side represents miRNAs with relatively lower expression in MIUC, whereas the right side represents those with relatively higher expression in MIUC. The top 20 miRNAs with the largest expression differences compared to NMIUC, along with their Q-values, are shown.Abbreviations: MIUC, muscle-invasive urothelial carcinoma; NMIUC, non-muscle-invasive urothelial carcinoma; log₂FC, log₂(fold change); RPM, reads per million; Q-value, adjusted p-value. [file 12885_2025_15284_MOESM1_ESM.pptx]

## Slide 1
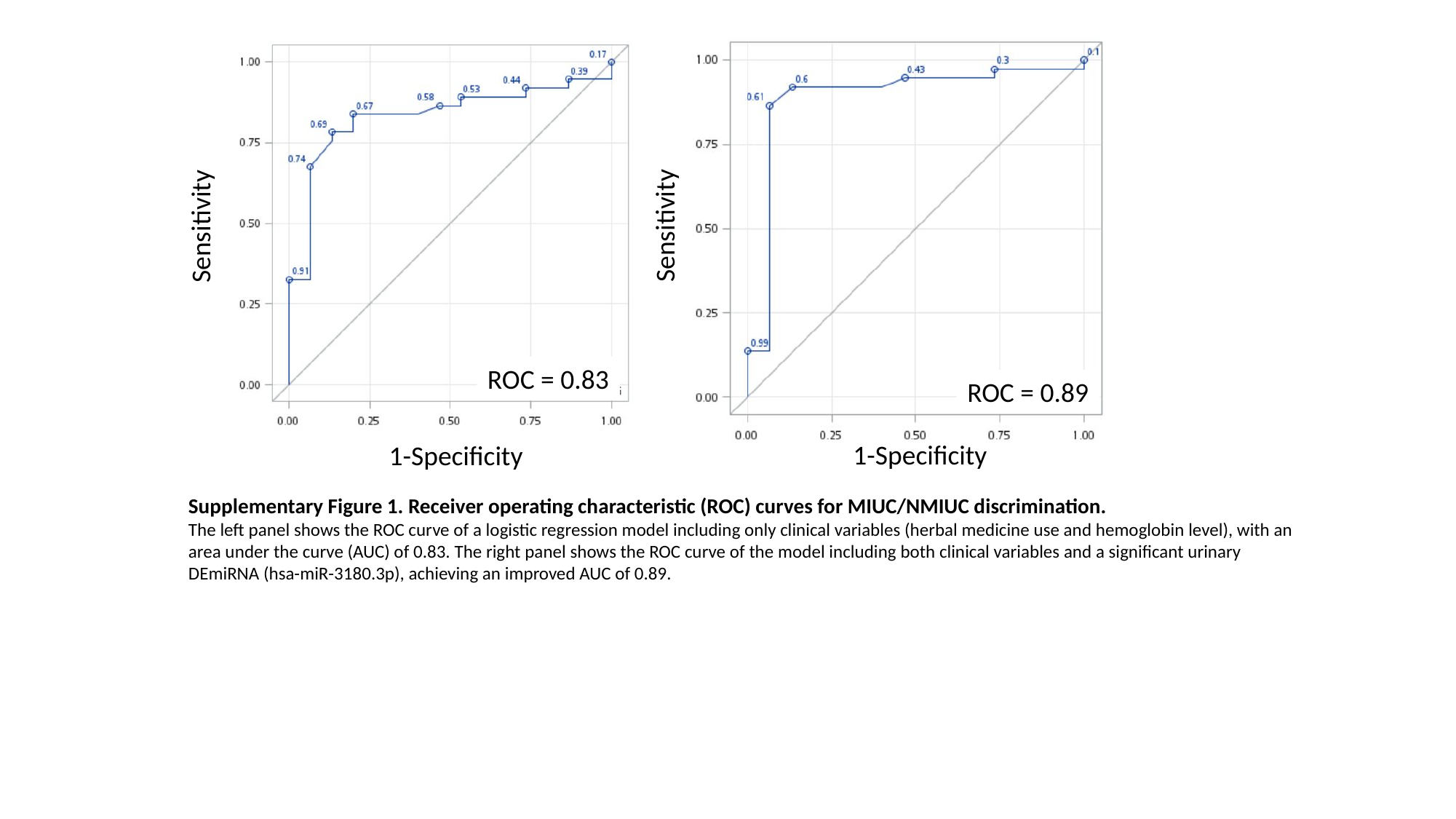

Sensitivity
Sensitivity
ROC = 0.83
ROC = 0.89
1-Specificity
1-Specificity
Supplementary Figure 1. Receiver operating characteristic (ROC) curves for MIUC/NMIUC discrimination.
The left panel shows the ROC curve of a logistic regression model including only clinical variables (herbal medicine use and hemoglobin level), with an area under the curve (AUC) of 0.83. The right panel shows the ROC curve of the model including both clinical variables and a significant urinary DEmiRNA (hsa-miR-3180.3p), achieving an improved AUC of 0.89.

## Slide 2
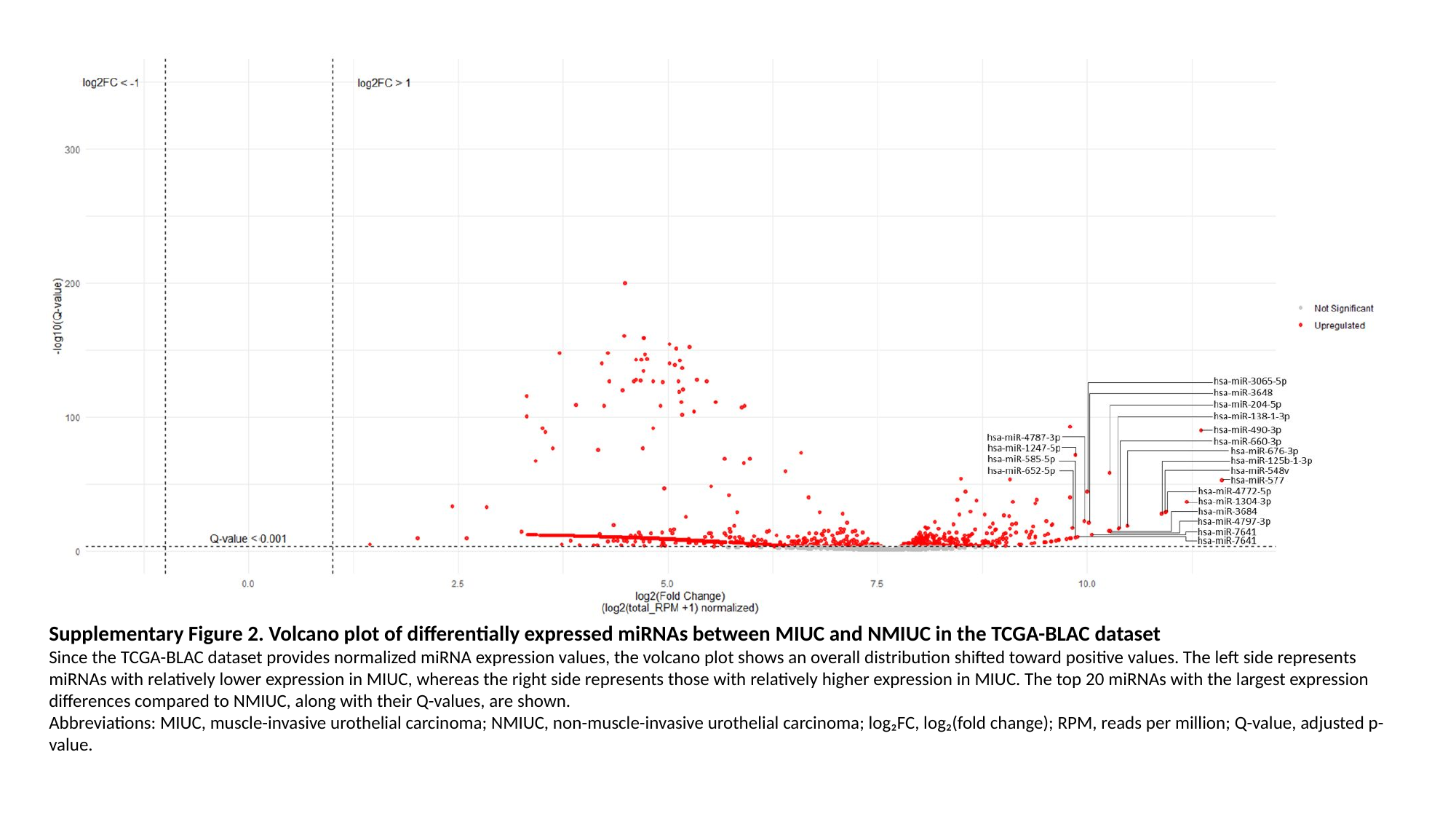

Supplementary Figure 2. Volcano plot of differentially expressed miRNAs between MIUC and NMIUC in the TCGA-BLAC dataset
Since the TCGA-BLAC dataset provides normalized miRNA expression values, the volcano plot shows an overall distribution shifted toward positive values. The left side represents miRNAs with relatively lower expression in MIUC, whereas the right side represents those with relatively higher expression in MIUC. The top 20 miRNAs with the largest expression differences compared to NMIUC, along with their Q-values, are shown.
Abbreviations: MIUC, muscle-invasive urothelial carcinoma; NMIUC, non-muscle-invasive urothelial carcinoma; log₂FC, log₂(fold change); RPM, reads per million; Q-value, adjusted p-value.
